# Evaluation of phenotype-driven gene prioritization methods for Mendelian diseases

**DOI:** 10.1093/bib/bbac188

**Published:** 2022-05-20

**Authors:** Julius O B Jacobsen, Catherine Kelly, Valentina Cipriani, Peter N Robinson, Damian Smedley

**Affiliations:** 1 William Harvey Research Institute, Charterhouse Square, Barts and the London School of Medicine and Dentistry Queen, Queen Mary University of London, EC1M 6BQ London, UK; 2 The Jackson Laboratory for Genomic Medicine, 10 Discovery Drive, Farmington, CT 06032, USA

**Keywords:** phenotype, rare disease, variant prioritization

## Abstract

Yuan *et al*. recently described an independent evaluation of several phenotype-driven gene prioritization methods for Mendelian disease on two separate, clinical datasets. Although they attempted to use default settings for each tool, we describe three key differences from those we currently recommend for our Exomiser and PhenIX tools. These influence how variant frequency, quality and predicted pathogenicity are used for filtering and prioritization. We propose that these differences account for much of the discrepancy in performance between that reported by them (15–26% diagnoses ranked top by Exomiser) and previously published reports by us and others (72–77%). On a set of 161 singleton samples, we show using these settings increases performance from 34% to 72% and suggest a reassessment of Exomiser and PhenIX on their datasets using these would show a similar uplift.

##  

We were pleased to see the recent publication in this journal on phenotype-driven gene prioritization methods for Mendelian disease [1]. This evaluation of the methods on two real clinical datasets (*N* = 305 and 209) will be welcomed by the clinical genetics community, especially as it is performed by an independent group that have not been involved in developing any of the assessed tools. However, we were surprised by the reported figures for our Exomiser tool, e.g. 15% and 26% of diagnoses identified as the top ranking hits in their two datasets compared with 38–58% for AMELIE, xRare and LIRICAL. As reported recently, we have used Exomiser to identify 77% of diagnoses as the top-ranking candidate for the 100 000 Genomes Project and this has led to its adoption in the Genomic Medicine Service of the UK National Health Service [2]. Similarly, for a retinal disease dataset 74% of diagnoses were ranked first by Exomiser [3] and a completely independent group from the Los Angeles Children Hospital reported a performance of 72% [4].

The authors state that default settings were used for each assessed software, but a closer inspection of the settings used by them when assessing Exomiser 12.1.0 revealed three key differences from those we routinely use. Here, we investigated whether these differences are likely to account for the above discrepancies. On a set of 161 diagnosed singleton cases from the 100 000 Genomes project (100KGP), using the same Exomiser version (12.1.0) they tested, we observe that 34% are identified as the top candidate with their settings versus 72% with ours. Incrementally reintroducing our settings to theirs highlights the differences these make:

(1) Including mode of inheritance (MOI) specific frequency filtering (0.1% for dominant or homozygous recessive modes, 2% for compound-heterozygous, recessive) increases performance to 44%. This is the recommended default in the version of Exomiser 12.1.0 they assessed and is closer to the minor allele frequency (MAF) filtering settings used for the higher performing tools in their hands such as LIRICAL and AMELIE. Note this filter, as used in the default recommendation, runs every possible MOI using appropriate MAF settings rather than restricting to preselected modes of inheritance that may not be known ahead of analysis.
(2) Including the failedFrequencyFilter further increases performance to 62%. This setting filters out any low-quality variants that are not flagged as PASS in the FILTER column of the VCF file. The settings Yuan *et al*. [1] used for LIRICAL and AMELIE did include filtering of non-PASS variants and this likely explains much of the improved performance they reported for these tools. Although this filter was documented in the example settings for Exomiser 12.1.0 it was not explicitly stated as a default recommendation. Since release 13 of Exomiser (September 2021) this is explicitly specified in the preset options.
(3) Using REVEL and MVP as more modern sources of predicted pathogenicity data than Polyphen2, MutationTaster and SIFT further increases performance to 72%. Again, although REVEL and MVP were available for the version of Exomiser tested (12.1.0), they were not clearly flagged as default settings but since release 13 they are now in the recommended preset options.

Figure 1 summarizes these results alongside the previously reported performance of Exomiser on clinical datasets.

**Figure 1:**
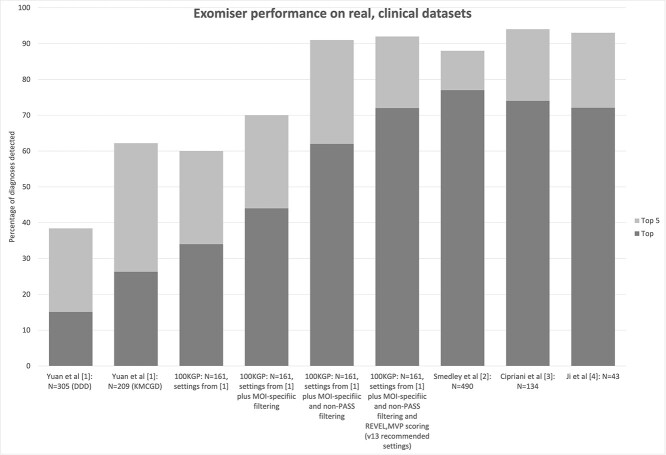
Performance of Exomiser on clinical datasets reported in previous studies [1–4] and here [100KGP] using various settings.

In conclusion, we recommend all users to use the preset options provided in Exomiser, or if they are altering configuration to make sure that the above three options are retained for optimal performance: e.g. the provided example VCF file (Pfeiffer.vcf.gz) and clinical data (pfeiffer-phenopacket.yml) will run with these recommended settings if simply run as java -jar exomiser-cli-13.0.1.jar—sample examples/pfeiffer-phenopacket.yml—vcf examples/Pfeiffer.vcf.gz—assembly hg19. PhenIX, another tool they assessed, runs as part of the Exomiser framework and will similarly benefit from using these recommended settings. We suspect that most, if not all, of the differences in performance the authors observe between xRare, AMELIE and LIRICAL and Exomiser/PhenIX are due to these variant filtering settings rather than the underlying gene prioritization algorithms they set out to evaluate. We would be very interested to see if this is the case in a future assessment of their datasets.

**Key Points**

- Variant filtering settings, as well as phenotype similarity approaches, are critical for the performance of phenotype-driven gene prioritization approaches.
- Mode of inheritance specific frequency and variant quality filtering alongside use of REVEL and MVP are the latest recommended options for Exomiser and PhenIX.
- Use of these latest recommended settings, versus those used in the study of Yuan *et al*., increased detection of the diagnosis as the top-ranked candidate from 34% to 72% in our hands.

## Notes

**Julius Jacobsen** is a researcher and software developer at Queen Mary University of London. He is responsible for the Exomiser software framework.

**Catherine Kelly** recently obtained a M.Sc. in Bioinformatics from Queen Mary University of London where her research focused on comparisons of variant prioritization software.

**Valentina Cipriani** is a Lecturer in Statistical Genomics at Queen Mary University of London where her work focuses on statistical approaches to understanding age related macular degeneration and gene-burden discovery approaches in rare disease.

**Peter Robinson** is a Professor of Computational Biology at the Jackson Laboratory where he leads a team developing essential tools for disease research such as the Human Phenotype Ontology.

**Damian Smedley** is a Professor of Computational Genomics at Queen Mary University of London. His team focuses on the use of clinical and model organism phenotype data to improve diagnosis and discovery in rare disease patients.
